# Obesity is a risk factor for poor response to treatment in early rheumatoid arthritis: a NORD-STAR study

**DOI:** 10.1136/rmdopen-2024-004227

**Published:** 2024-04-04

**Authors:** Violetta Dubovyk, Georgios K Vasileiadis, Tahzeeb Fatima, Yuan Zhang, Meliha Crnkic Kapetanovic, Alf Kastbom, Milad Rizk, Annika Söderbergh, Sizheng Steven Zhao, Ronald F van Vollenhoven, Merete Lund Hetland, Espen A Haavardsholm, Dan Nordström, Michael T Nurmohamed, Bjorn Gudbjornsson, Jon Lampa, Mikkel Østergaard, Marte Schrumpf Heiberg, Tuulikki Sokka-Isler, Gerdur Gröndal, Kristina Lend, Kim Hørslev-Petersen, Till Uhlig, Anna Rudin, Cristina Maglio

**Affiliations:** 1 Department of Rheumatology and Inflammation Research, University of Gothenburg, Gothenburg, Sweden; 2 Department of Clinical Science, Skåne University Hospital Lund, Lund, Skåne, Sweden; 3 Department of Biomedical and Clinical Sciences, Linköping University, Linkoping, Sweden; 4 Rheumatology Clinic, Västmanlands Hospital, Vasteras, Sweden; 5 Department of Rheumatology, Örebro University Hospital, Orebro, Sweden; 6 Centre for Epidemiology Versus Arthritis, Division of Musculoskeletal and Dermatological Science, The University of Manchester, Manchester, UK; 7 Department of Rheumatology, Amsterdam Rheumatology and Immunology Center, Amsterdam, The Netherlands; 8 Rheumatology Unit, Department of Medicine, Karolinska Institute, Stockholm, Sweden; 9 Copenhagen Center for Arthritis Research, Center for Rheumatology and Spine Diseases, Rigshospitalet Glostrup, Glostrup, Denmark; 10 Department of Clinical Medicine, Faculty of Health Sciences, University of Copenhagen, Copenhagen, Denmark; 11 Centre for treatment of Rheumatic and Musculoskeletal Diseases, Diakonhjemmet Hospital, Oslo, Norway; 12 Faculty of Medicine, University of Oslo, Oslo, Norway; 13 Department of Medicine and Rheumatology, Helsinki University Central Hospital, Helsinki, Uusimaa, Finland; 14 Amsterdam Rheumatology and Immunology center, Amsterdam, The Netherlands; 15 Faculty of Medicine, University of Iceland, Reykjavik, Iceland; 16 Centre for Rheumatology Research, Landspitali University Hospital, Reykjavik, Iceland; 17 Department of Medicine, Jyväskylä Central Hospital, University of Eastern Finland, Jyväskylä, Finland; 18 Danish Hospital for Rheumatic Diseases, University Hospital of Southern Denmark, Sonderborg, Denmark; 19 Department of Regional Health Research, University of Southern Denmark, Odense, Denmark

**Keywords:** Treatment, Rheumatoid Arthritis, Risk Factors

## Abstract

**Objective:**

This report from the NORD-STAR (Nordic Rheumatic Diseases Strategy Trials and Registries) trial aimed to determine if obesity is associated with response to conventional and biological antirheumatic treatment in early rheumatoid arthritis (RA).

**Methods:**

This report included 793 participants with untreated early RA from the randomised, longitudinal NORD-STAR trial, all of whom had their body mass index (BMI) assessed at baseline. Obesity was defined as BMI ≥30 kg/m^2^. All participants were randomised 1:1:1:1 to one of four treatment arms: active conventional treatment, certolizumab-pegol, abatacept and tocilizumab. Clinical and laboratory measurements were performed at baseline and at 8, 12, 24 and 48-week follow-up. The primary endpoint for this report was response to treatment based on Clinical Disease Activity Index (CDAI) and Simple Disease Activity Index (SDAI) remission and Disease Activity Score with 28 joints using C-reactive protein (DAS28-CRP) <2.6 stratified by BMI.

**Results:**

Out of 793 people included in the present report, 161 (20%) had obesity at baseline. During follow-up, participants with baseline obesity had higher disease activity compared with those with lower BMI, despite having similar disease activity at baseline. In survival analyses, obesity was associated with a lower likelihood of achieving response to treatment during follow-up for up to 48 weeks (CDAI remission, HR 0.84, 95% CI 0.67 to 1.05; SDAI, HR 0.77, 95% CI 0.62 to 0.97; DAS28-CRP <2.6, HR 0.78, 95% CI 0.64 to 0.95). The effect of obesity on response to treatment was not influenced by the treatment arms.

**Conclusion:**

In people with untreated early RA followed up for up to 48 weeks, obesity was associated with a lower likelihood of good treatment response, irrespective of the type of randomised treatment received.

**Trial registration number:**

NCT01491815.

**WHAT IS ALREADY KNOWN ON THIS TOPIC**

- Obesity is a risk factor for rheumatoid arthritis (RA) and has been associated with poor treatment response.
- However, the association between obesity and response to treatment in early RA is less clear, especially for treatment-naïve patients who start biological antirheumatic drugs.

**WHAT THIS STUDY ADDS**

- By studying a large longitudinal cohort of people with untreated early RA followed up for up to 48 weeks, we showed that obesity at diagnosis was a risk factor for poor response to treatment.
- The association between obesity and treatment response was independent of the treatment arms, which included both conventional antirheumatic drugs and three biological drugs with different mechanisms of action.

**HOW THIS STUDY MIGHT AFFECT RESEARCH, PRACTICE OR POLICY**

- People with newly diagnosed RA who also have obesity should be carefully monitored as they are at high risk of treatment failure.

## Introduction

Rheumatoid arthritis (RA) is a chronic inflammatory autoimmune disease primarily affecting the joints. Left untreated, RA may lead to joint destruction, systemic complications, physical disability and reduced life expectancy.^1^ Several options are available to treat RA, including conventional synthetic disease-modifying antirheumatic drugs (csDMARDs), biological disease-modifying antirheumatic drugs (bDMARDs) and targeting synthetic disease-modifying antirheumatic drugs (DMARDs).^2^ Remission is the treatment goal in RA but the response to treatment is highly variable, and not all patients achieve low disease activity or clinical remission.^3–6^ Currently, there is no way to precisely predict which patient with RA is more likely to respond to a certain treatment.^4 6–8^ In fact, the use of levels of circulating autoantibodies, inflammation parameters such as C-reactive protein (CRP) and erythrocyte sedimentation rate (ESR), or composite clinical scores such as Clinical Disease Activity Index (CDAI) or Disease Activity Score with 28 joints using C-reactive protein (DAS28-CRP) as predictors, has proven to be insufficient in directing clinicians in the choice of DMARDs.^7–9^ Treatment failure in RA is associated with an increased risk of long-term complications and reduced quality of life for patients, as well as increased economic burden for the society.^10^

Obesity is an established risk factor for developing RA.^11–14^ Moreover, people with RA that have overweight or obesity more often need combination therapy and may have lower chance to achieve low disease activity or remission compared with normal-weight people, although this is debated especially for those treated with bDMARDs.^15–20^ Whether obesity affects the response to antirheumatic treatment in early RA is less clear as both similar and lower chances to achieve remission have been reported for people with obesity compared with those with lower body mass index (BMI).^21–24^ Furthermore, the effect of BMI on response to different bDMARDs has never been studied in people with newly diagnosed RA.

Here, we aimed to determine if obesity at baseline affects disease activity and response to csDMARDs and bDMARDs up to 48-week follow-up in a well-characterised cohort of treatment-naïve participants with early RA, the Nordic Rheumatic Diseases Strategy Trials and Registries (NORD-STAR).^25–27^ NORD-STAR is a randomised longitudinal trial designed to compare efficacy and safety of three bDMARDs with different targets versus active conventional treatment in early RA. This study provides a unique opportunity to study the association between BMI and response to treatment in early RA.

## Patients and methods

### Patients and study design

**Figure 1:**
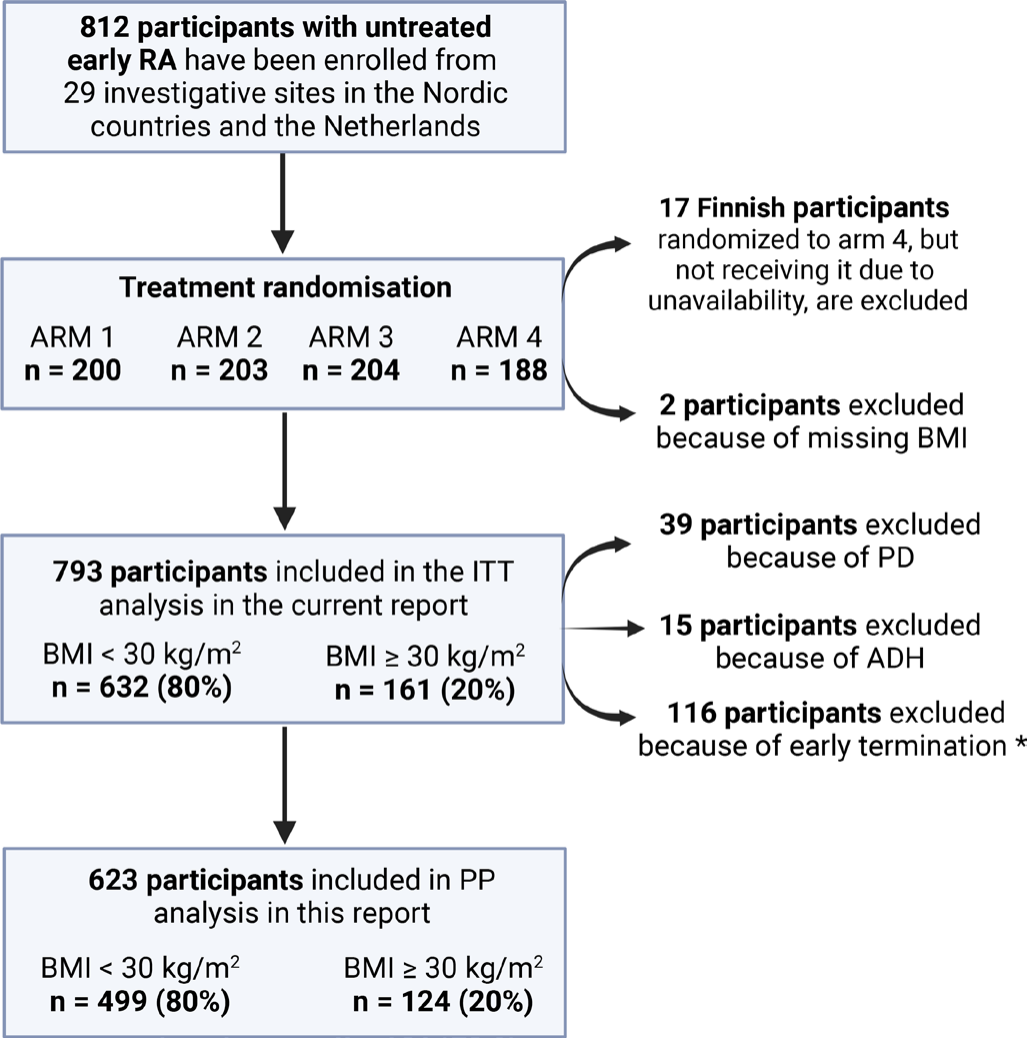
Study design of the present report. Early termination because of abnormal laboratory results (which ruled out continuation of the study drug as determined by the investigator), lack of efficacy, death or other reasons. Arm 1—active conventional treatment (ACT); arm 2—methotrexate (MTX)+certolizumab; arm 3— MTX+abatacept; arm 4—MTX+tocilizumab. ADH, failure to adhere to the protocol; BMI, body mass index; ITT analysis, intention-to-treat analysis; PD, protocol deviation, PP analysis, per-protocol analysis; RA, rheumatoid arthritis.

NORD-STAR is a multicentre, randomised, open-label, blinded-assessor, phase IV longitudinal trial, designed to compare efficacy and safety of active conventional antirheumatic treatment versus three different bDMARDs in participants with untreated early RA.^26 28^ A total of 812 adult participants were enrolled at 29 investigative sites in Sweden, Norway, Denmark, Finland, Iceland and the Netherlands (figure 1).

People newly diagnosed with RA between 2012 and 2018 according to the American College of Rheumatology (ACR)/European Alliance of Associations for Rheumatology (EULAR) 2010 classification criteria were assessed for eligibility.^29^ Inclusion criteria were symptom duration <24 months, ≥18 years of age, DAS28-CRP >3.2, ≥2 swollen joints and ≥2 tender joints (based on 66/68 joint count). Participants also had to fulfil at least one of the following three criteria: rheumatoid factor (RF) positivity, or anti-citrullinated protein antibody (ACPA) positivity or CRP ≥10 mg/L. Exclusion criteria included, among others, previous DMARD therapy for rheumatic diseases, current active inflammatory joint disease other than RA, chronic pain syndrome, active infection of any kind, history of blood, liver or kidney disease or cancer, alcohol or drug abuse.^25^

### Randomisation and intervention

At baseline, study participants were randomised 1:1:1:1 to one of four treatment arms. All arms included methotrexate up to 25 mg/week. In arm 1, called active conventional treatment, participants received sulfasalazine (2 g/day), hydroxychloroquine (35 mg/kg/week or 200 mg/day) and intra-articular glucocorticoids in the swollen joints (maximally four joints and 80 mg per visit) if needed (arm 1A, used in Denmark and Finland) or orally administered prednisolone (arm 1B in Iceland, the Netherlands, Norway and Sweden). Participants in arm 2 received a tumour necrosis factor (TNF) inhibitor, certolizumab-pegol in dose 200 mg every other week subcutaneously (400 mg at 0, 2 and 4 weeks); those in arm 3 received abatacept (cytotoxic T-lymphocyte-associated molecule-4 immunoglobulin), 125 mg/week subcutaneously, whereas participants in arm 4 received tocilizumab (interleukin 6 receptor inhibitor) 8 mg/kg every 4 weeks intravenously or 162 mg/week subcutaneously. During the entire study, the use of non-steroidal anti-inflammatory drugs was allowed. Stratification for randomisation was performed by country, sex and ACPA status. The numbers of participants in treatment arms 1, 2, 3 and 4 were 217, 203, 204 and 188, respectively.

### Population, measurements and outcomes of the current report

Out of 812 NORD-STAR study participants, 17 Finnish participants have been excluded from the current report because they were randomised to arm 4 (tocilizumab+MTX) but did not receive treatment due to unavailability, as also shown in previous reports from the NORD-STAR study (figure 1).^26 27^ Moreover, two people have been excluded from the current report as height and weight measurements at baseline were missing. Out of 793 study participants included in the current report (intention-to-treat protocol, ITT), 200 were randomised to arm 1, 203 to arm 2, 203 to arm 3 and 187 arm 4. In the per-protocol population (n=623, figure 1), we have excluded study participants who deviated from protocol or did not adhere to protocol or had an early termination (due to abnormal laboratory results, lack of efficacy, death and other reasons).

The following cut-offs were used to define BMI classes: under or normal weight as BMI <25 kg/m^2^, overweight as BMI ≥25 and <30 kg/m^2^, and obesity as BMI ≥30 kg/m^2^.^30^ Clinical and laboratory measurements were performed at baseline and at 8, 12, 24 and 48-week follow-up. The relative delta change between baseline and follow-up values were calculated as: (follow-up value – baseline value)/baseline value. The primary clinical endpoint for the NORD-STAR study was remission at 24 weeks according to CDAI (CDAI ≤2.8). Primary outcomes for the current report were the differences in CDAI remission (CDAI ≤2.8) over time up to 48 weeks between participants with obesity and without obesity. Achievement of DAS28-CRP <2.6 and Simple Disease Activity Index (SDAI) remission (SDAI ≤3.3) were considered as the co-primary outcomes. The impact of treatment randomisation was assessed on all primary study outcomes. Achievement of DAS28-CRP low disease activity (DAS28-CRP≤3.2) and sustained response to treatment (defined as achieving CDAI and SDAI remission or DAS28-CRP <2.6 at two or more consecutive visits until end of follow-up) were shown as secondary outcomes. For the purpose of the analyses, participants will be considered as responders and non-responders for any dichotomous outcomes.

### Analysis and statistical methodology

Differences in categorical variables were assessed using the χ^2^ test, whereas differences between continuous variables were assessed using the t-test. Regression analyses, using the fixed effect models, were employed to assess the associations between BMI (as dichotomous variable) and response to treatment. Models were further adjusted for age, sex, Patient Pain assessment by Visual Analogue Scale (VAS), ACPA status, current smoking, treatment randomisation and additionally for DAS28-CRP at baseline when specified. Interaction terms were calculated as treatment arms×obesity as well as tocilizumab administration (intravenous and subcutaneous administration)×obesity (as a categorical variable).

Survival analyses were performed to determine if obesity was associated with response to treatment during the follow-up period. Time to achieve the outcome was assessed by Kaplan-Meier estimates of cumulative incidence rates and compared between BMI groups by log-rank test. HRs and corresponding 95% CIs for the response to treatment were calculated with Cox proportional hazard models. HRs are presented as both unadjusted and adjusted for sex, baseline age, Patient Pain by VAS and DAS28-CRP in model 1, and additionally for current smoking, ACPA and treatment randomisation in model 2. All analyses in the current report are ITT based, unless otherwise specified (n=793, figure 1).

## Results

### Baseline characteristics

Out of 793 participants with untreated early RA from the NORD-STAR study included in the current report, 161 (20%) had obesity at baseline (table 1). Inflammatory parameters, swollen and tender joint counts were similar between participants with obesity and the rest of the cohort. However, patients’ global and pain assessments by VAS were higher in participants with obesity versus those without obesity (Patient Global by VAS 62±22 vs 57±24; Patient Pain by VAS 61±24 vs 56±24, respectively, both p=0.01, table 1), and participants with obesity had a slightly higher DAS28-CRP score (5.2±1.0 and 5.0±1.1, p=0.03, table 1). Other scores of disease activity, such as SDAI and CDAI, were not significantly different between the two groups. The baseline characteristics of participants included in the present report stratified by treatment arm and BMI are shown in online supplemental table 1.

10.1136/rmdopen-2024-004227.supp1 Supplementary data

**Table 1:** Baseline characteristics of study participants from the NORD-STAR cohort stratified by BMI=30 kg/m^2^

Characteristics	BMI (kg/m^2^)	
	<30	≥30
n (%)	632 (80)	161 (20)
Age, years	54±15	55±12
Women, n (%)	430 (68)	117 (73)
BMI	24±3	34±4***
Current smokers, n (%)	140 (22)	33 (21)
Ever smokers, n (%)	369 (58)	105 (65)
RF positive, n (%)	472 (75)	120 (75)
ACPA positive, n (%)	516 (82)	133 (83)
Symptom duration, days	202±158	214±185
Time since diagnosis, days	13±24	16±38
ESR, mm/hour	32±24	34±24
CRP, mg/L	21±29	19±22
SJC28	8±5	8±5
SJC66	11±7	10±7
TJC28	9±6	10±6
TJC68	16±11	16±10
Patient Global by VAS (0–100 mm)	57±24	62±22*
Patient Pain by VAS (0–100 mm)	56±24	61±24*
Physician Global by VAS (0–100 mm)	49±19	52±17
DAS28-ESR	5.3±1.2	5.5±1.0
DAS28-CRP	5.0±1.1	5.2±1.0*
SDAI	30±13	31±11
CDAI	28±12	29±11
Treatment arms		
Arm 1, n (%)	155 (25)	45 (28)
Arm 2, n (%)	165 (26)	38 (24)
Arm 3, n (%)	164 (26)	39 (24)
Arm 4, n (%)	148 (23)	39 (24)

Data are shown as means±SD for continuous variables and as number (percentage) for categorical variables. Missing data as follows: n=3 for symptom duration, n=2 for time since diagnosis, n=7 for CDAI, n=7 for Patient Global by VAS (0–100 mm), n=7 for Physician Global by VAS (0–100 mm), n=2 for CRP, n=9 for SDAI, n=205 both for ESR and DAS28-ESR.

Independent samples t-test was used for continuous variables to test whether the population means of two groups are equal or not. Differences between the two groups were analysed using a χ^2^ test for categorical variables. *p<0.05, **p<0.01, ***p<0.001.

Arm 1—active conventional treatment (ACT); arm 2—methotrexate (MTX)+certolizumab; arm 3—MTX+abatacept; arm 4—MTX+tocilizumab.

ACPA, anti-cyclic citrullinated peptide; BMI, body mass index; CDAI, Clinical Disease Activity Index; CRP, C-reactive protein; DAS28-CRP, Disease Activity Score with 28 joints using C-reactive protein; DAS28-ESR, Disease Activity Score with 28 joint using erythrocyte sedimentation rate; ESR, erythrocyte sedimentation rate; RF, rheumatoid factor; SDAI, Simplified Disease Activity Index; SJC, swollen joint count; TJC, tender joint count; VAS, Visual Analogue Scale.

### Disease activity and response to treatment over time

**Figure 2:**
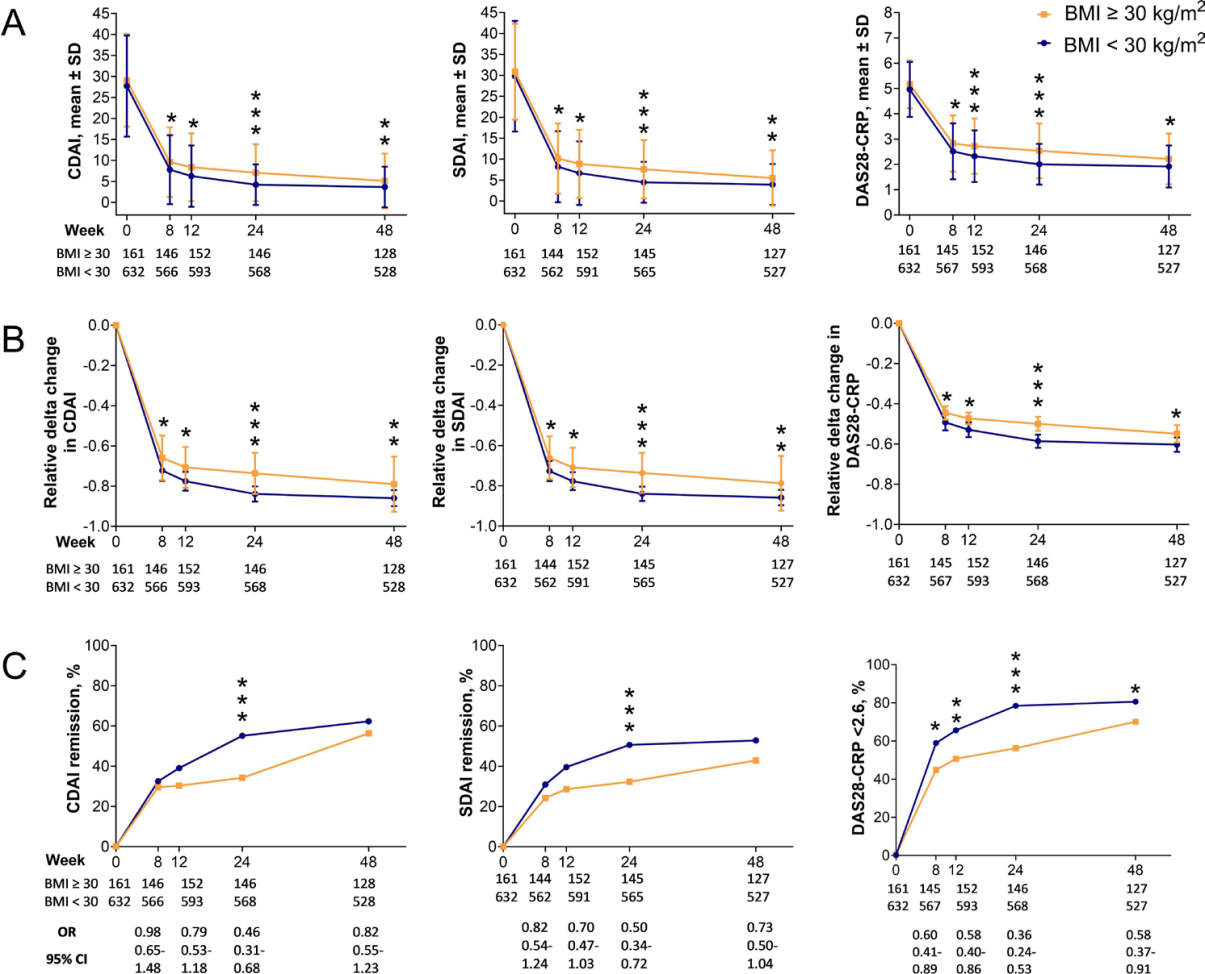
Disease activity scores and response to treatment percentage over time stratified by BMI=30 kg/m^2^. Mean values (A), level changes (B) and remission scores (C) for CDAI, SDAI and DAS28-CRP <2.6. Markers of disease activity are shown as mean values and SD. Relative delta changes in markers of disease activity are shown as mean and 95% CI. The relative delta changes between baseline and follow-up values were calculated as: (follow-up value – baseline value)/baseline value. Response to treatment is shown as % of participants who achieved response to treatment at certain time points during follow-up. P values for continuous variables have been calculated by linear regression analysis adjusted for sex, baseline age, current smoking, Patient Pain by VAS, DAS28-CRP, ACPA and treatment randomisation. P values for remission rates have been calculated by logistic regression analysis adjusted for sex, baseline age, current smoking, Patient Pain by VAS, DAS28-CRP, ACPA and treatment randomisation. *p<0.05, **p<0.01, ***p<0.001. ACPA, anti-cyclic citrullinated peptide; BMI, body mass index; CDAI, Clinical Disease Activity Index; DAS28-CRP, Disease Activity Score with 28 joints using C-reactive protein; SDAI, Simplified Disease Activity Index; VAS, Visual Analogue Scale (0–100 mm).

**Figure 3:**
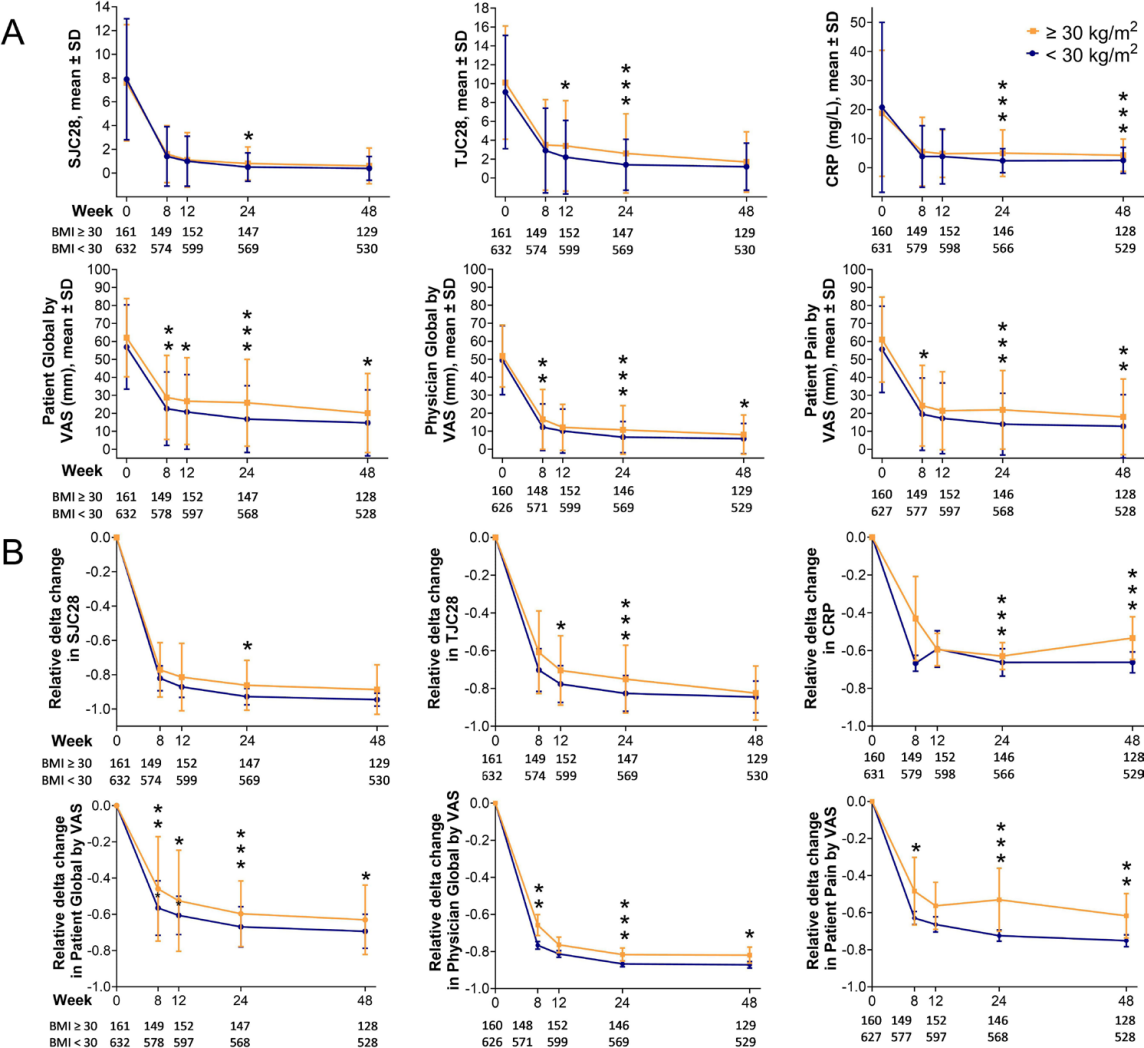
Disease activity markers over time in study participants stratified by BMI=30 kg/m^2^. Mean values (A) and level changes (B) for SJC28, TJC28, CRP, Patient Global by VAS (0–100 mm), Physician Global by VAS (0–100 mm) and Patient Pain by VAS (0–100 mm). Markers of disease activity are shown as mean values and SD. Relative delta changes in markers of disease activity are shown as mean and 95% CI. The relative delta changes between baseline and follow-up values were calculated as: (follow-up value – baseline value)/baseline value. P values have been calculated by linear regression analysis adjusted for sex, baseline age, current smoking, Patient Pain by VAS, DAS28-CRP, ACPA and treatment randomisation. *p<0.05, **p<0.01, ***p<0.001. ACPA, anti-cyclic citrullinated peptide; BMI, body mass index; CRP, C-reactive protein; DAS28-CRP, Disease Activity Score with 28 joints using C-reactive protein; SJC, swollen joint count; TJC, tender joint count; VAS, Visual Analogue Scale of pain (0–100 mm).

First, we wanted to determine if participants with obesity had higher disease activity after antirheumatic treatment initiation compared with people with BMI <30 kg/m^2^. Overall, participants with baseline obesity had higher disease activity and smaller reduction in disease activity scores over time from week 8 throughout week 48 compared with participants with BMI <30 kg/m^2^ as assessed by CDAI, SDAI and DAS28-CRP (figure 2A,B). Moreover, the odds of achieving CDAI and SDAI remission as well as DAS28-CRP <2.6 were lower in participants with obesity versus those with BMI <30 kg/m^2^ (figure 2C). This difference was more evident at week 24 compared with week 48 (CDAI remission, OR 0.46, 95% CI 0.31 to 0.68 at 24-week follow-up vs OR 0.82, 95% CI 0.55 to 1.23 at 48-week follow-up; SDAI remission, OR 0.50, 95% CI 0.34 to 0.72 at 24-week follow-up vs OR 0.73, 95% CI 0.50 to 1.04 at 48-week follow-up; DAS28-CRP <2.6, OR 0.36, 95% CI 0.24 to 0.53 at 24-week follow-up vs OR 0.58, 95% CI 0.37 to 0.91 at 48-week follow-up, figure 2C). When looking at different markers of disease activity from week 8 to week 48, higher tender joint counts, CRP and assessment scores of disease activity and pain were observed in participants with obesity compared with the rest of the cohort (figure 3A). Participants with obesity at baseline also had less reduction in swollen and tender joint counts, CRP and assessment scores of disease activity and pain after treatment initiation compared with the rest of the cohort (figure 3B).

### Response to treatment incidence

**Figure 4:**
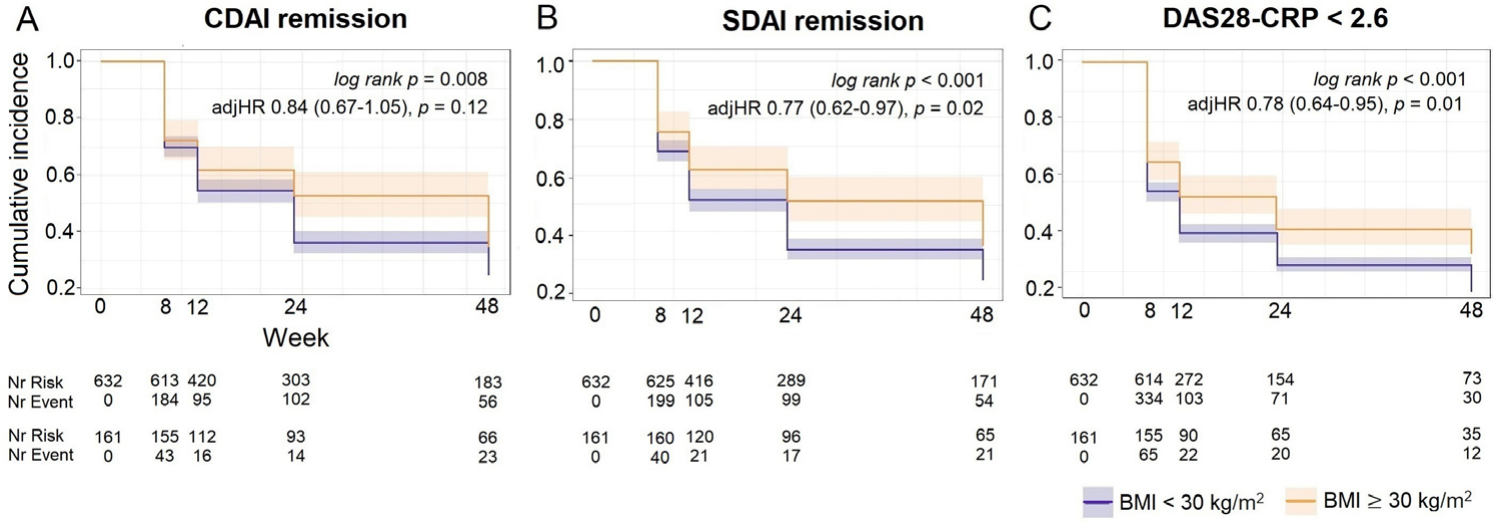
Kaplan-Meier curves for response to treatment according to BMI=30 kg/m^2^. CDAI remission (A); SDAI remission (B); DAS28-CRP <2.6 (C). Log-rank p values together with HRs and p values adjusted for sex, baseline age, current smoking, Patient Pain by VAS, DAS28-CRP, ACPA and treatment randomisation. ACPA, anti-cyclic citrullinated peptide; BMI, body mass index; CDAI, Clinical Disease Activity Index; DAS28-CRP, Disease Activity Score with 28 joints using C-reactive protein; SDAI, Simplified Disease Activity Index; VAS, Visual Analogue Scale of pain.

Then, we went on to determine if obesity affected the chances to achieve response to treatment in people with early RA. Obesity was associated with a lower likelihood of achieving CDAI remission in participants with early RA followed up for up to 48 weeks after initiation of treatment (figure 4A). In total, 49% of the participants with obesity (n=81) achieved CDAI remission compared with 57% of the participants with BMI <30 kg/m^2^ (n=365) after 48 weeks of treatment. Similar differences were observed for the achievement of SDAI remission and DAS28-CRP <2.6 (figure 4B,C). In survival analyses, participants with obesity at baseline were less likely to achieve response to treatment compared with those with BMI <30 kg/m^2^ (CDAI remission, adjusted HR 0.84, 95% CI 0.67 to 1.05; SDAI remission, HR 0.77, 95% CI 0.62 to 0.97; DAS28-CRP <2.6, HR 0.78, 95% CI 0.64 to 0.95; figure 4 and online supplemental table 2).

### BMI-treatment interaction

**Figure 5:**
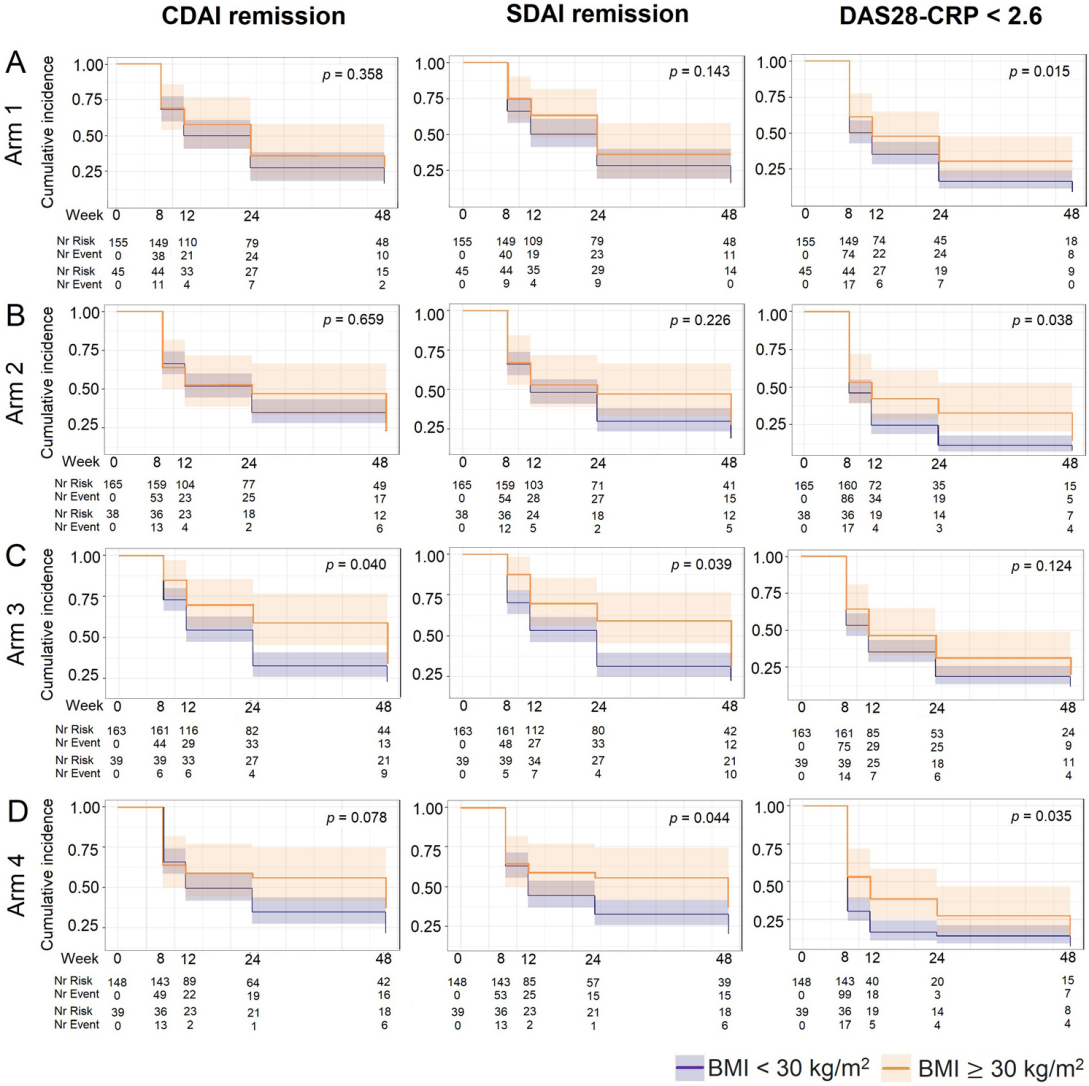
Kaplan-Meier curves for CDAI, SDAI remission, DAS28-CRP <2.6 stratified by treatment arms. Arm 1 —active conventional treatment (ACT; A); arm 2—methotrexate (MTX)+certolizumab (B); arm 3— MTX+abatacept (C); arm 4—MTX+tocilizumab (D). BMI, body mass index; CDAI, Clinical Disease Activity Index; DAS28-CRP, Disease Activity Score with 28 joints using C-reactive protein; SDAI, Simplified Disease Activity Index.

The BMI-treatment interaction term was not significant for the incidence of CDAI, SDAI remission and DAS28-CRP <2.6 up to 48-week follow-up (p=0.45, p=0.88, p=0.64 respectively). In all treatment arms, the percentages of participants achieving CDAI and SDAI remission, and DAS28-CRP <2.6 were lower in participants with obesity compared with those with BMI <30 kg/m^2^ (online supplemental figure 1). As shown in figure 5 and online supplemental table 3, participants with RA and obesity had overall lower likelihood of achieving CDAI and SDAI remission and DAS28-CRP <2.6 compared with the rest of the cohort, regardless of treatment arm.

Participants randomised to arm 4 received either subcutaneous tocilizumab at a standard dose or intravenous tocilizumab at a dose based on body weight. To determine if the worse response to treatment in participants with obesity is due to the fact that they receive inadequate drug doses in relation to their body weight, we added obesity×tocilizumab administration (intravenously vs subcutaneously) term in the analysis for arm 4. The obesity-tocilizumab administration interaction term was not significant with regard to the achievement of CDAI and SDAI remission as well as DAS28-CRP <2.6 (p=0.95, p=0.79, p=0.99, respectively).

### Sensitivity analyses

Finally, we have performed a series of sensitivity analyses to confirm our findings. When looking at DAS28-CRP low disease activity as a less stringent definition of response to treatment, we have seen that participants with baseline obesity also had a lower likelihood to achieve DAS28-CRP low disease activity compared with participants with BMI <30 kg/m^2^ (online supplemental figure 2). The HR for achieving DAS28-CRP low disease activity was 0.78 (95% CI 0.64 to 0.95) for participants with obesity compared with non-obese participants (online supplemental table 4). Participants with obesity had also a lower chance to achieve sustained remission as measured by CDAI (HR 0.68, 95% CI 0.50 to 0.93), SDAI (HR 0.63, 95% CI 0.46 to 0.87) and sustained DAS28-CRP <2.6 (HR 0.73, 95% CI 0.58 to 0.92) compared with participants with lower BMI (online supplemental figure 3 and online supplemental table 5).

We have also determined if having a BMI ≥25 kg/m^2^ was associated with lower treatment response in early RA. In total, 420 out of 793 participants had overweight or obesity (BMI ≥25 kg/m^2^).^28^ Participants with BMI ≥25 kg/m^2^ had a lower likelihood of achieving DAS28-CRP <2.6 compared with those with normal weight, and similar Kaplan-Maier curves are seen for CDAI and SDAI remission (online supplemental figure 4). After adjustment, no significant difference in the HR for treatment response was seen in participants with overweight and obesity compared with those with BMI <25 kg/m^2^ (online supplemental table 6). Kaplan-Meier curves for the different BMI classes (under/normal weight, overweight and obesity) showed a decreasing likelihood of achieving response to treatment as the BMI increased, although the reduction in the HR for treatment response was significant only for BMI ≥30 kg/m^2^ compared with BMI <25 kg/m^2^ (online supplemental figure 5, online supplemental table 7).

Per-protocol analyses of remission during follow-up and incidence of remission in participants with obesity versus those with BMI <30 kg/m^2^ are shown in online supplemental figures 6 and 7 and online supplemental table 8. Overall, the per-protocol analysis, as the ITT analysis, showed that participants with obesity had lower chances to achieve response to treatment for all disease activity scores.

## Discussion

This report from the longitudinal randomised trial NORD-STAR showed that participants with untreated early RA and obesity had worse response to antirheumatic treatment compared with participants with lower BMI. This association was not influenced by the treatment arms, which included both csDMARDs and bDMARDs. Throughout follow-up for up to 48 weeks, participants with baseline obesity had higher disease activity after treatment initiation compared with participants with lower BMI, despite similar disease activity markers at baseline.

Before treatment initiation, NORD-STAR participants with early RA and obesity had slightly higher self-assessment scores for disease activity and pain compared with the rest of the cohort, despite having similar inflammatory markers and number of swollen and tender joints. Decreased quality of life and higher pain rating scales are frequent in obesity, with or without a concomitant chronic condition.^21 31^ Chronic pain is also common in people with obesity^32^; however, it should be noted that chronic pain syndrome was one of the exclusion criteria of the NORD-STAR study. The discrepancy between self-assessment scores and objective markers of disease activity may be also due to the difficult clinical assessment of swollen joints in participants with obesity.^33 34^ However, when synovitis are assessed by magnetic resonance, participants with obesity do not have a higher swollen joint count compared with those with lower BMI.^35^

During follow-up for up to 48 weeks after DMARD treatment initiation, participants with baseline obesity had higher disease activity scores compared with the rest of the cohort. When looking at the different components of the disease activity scores, participants with obesity had not only higher patients’ and physicians’ assessments scores but also higher tender joint counts and CRP values compared with participants with lower BMI. As for baseline values, the number of swollen joints was similar between patients with high and low BMI throughout the entire follow-up, which might be a consequence of the challenging clinical assessment of synovitis in people with obesity.^33 34^

Previous studies have reported a lower likelihood for people with RA having obesity to reach disease remission after treatment.^23 36 37^ A meta-analysis including studies on people with RA at any disease stage treated with bDMARDs has reported decreased rates for remission in participants with obesity.^16^ However, whether obesity affected the chances to achieve remission in untreated early RA was less clear. Both similar and lower chances to achieve remission have been recently reported for early RA people with obesity compared with those with lower BMI.^21 22^ A multicentre prospective study has reported that early RA participants with obesity had a lower likelihood of sustained remission during a 3-year follow-up; however, only 3% of this cohort was treated with bDMARDs.^24^ In this report, we show that obesity negatively affected the chance to achieve response to treatment in a large cohort of treatment-naïve participants randomised to four different treatments, including both csDMARDs and bDMARDs. Various sensitivity analyses have been performed, confirming that participants with early RA and high BMI had a lower likelihood to achieve remission after treatment to participants with lower BMI. This difference is clinically relevant and is more evident at 24-week follow-up, where participants with obesity had 50% lower odds to respond to treatment compared with participants with lower BMI.

We also looked at the impact of obesity on remission according to the four treatment arms, ranging from csDMARDs to three different bDMARDs with different mechanisms of action. We found no interaction between obesity and the treatment arms in terms of the likelihood of achieving remission up to 48-week follow-up. When looking at the different treatment arms, participants with obesity had an overall lower incidence of remission compared with participants with lower BMI. However, the number of participants in each treatment group is around 200 people, which makes it difficult to draw any conclusion regarding the impact of BMI on remission in each group.

The reasons for lower odds of achieving response to treatment in people with RA having obesity are not completely clear. NORD-STAR participants with obesity had shown higher self-assessed pain scores from baseline until the end of follow-up compared with those with lower BMI, which have contributed to the lower response to treatment. Nevertheless, the difference in self-assessed pain between participants with and without obesity is mild and of uncertain clinical relevance, even if significant, and does not explain the higher CRP levels seen in participants with obesity at 24-week and 48-week follow-up. Inflammatory parameters such as CRP are known to be mildly elevated in obesity, as a marker of low-grade subclinical systemic inflammation^38 39^; however, in our cohort, participants with obesity did not have higher CRP levels compared with participants with lower BMI before treatment initiation. Another possible explanation is that people with RA having obesity might be getting inadequate doses of DMARDs in relation to their body weight and therefore might be undertreated.^40^ In a sensitivity analysis, we have looked at the impact of different administrations of tocilizumab, either intravenously based on body weight or subcutaneously by standard dose. The interaction between mode of administration and obesity was not significant. However, this analysis was possibly underpowered in terms of sample size and further studies in bigger population are warranted to determine if administration of DMARDs based on bodily weight is more effective than standard doses in people with both RA and obesity. How the lower chances of achieving remission after treatment affect the long-term natural history of RA in people with obesity is unclear. People with RA and obesity seem to have lower radiographic progression compared with those with lower body weight,^41^ despite having lower likelihood to achieve disease remission. On the other hand, obesity is a risk factor for the development of interstitial lung disease in people with RA independently of other factors, which advocates for tighter control and intensification of treatment in this group of patients.^42 43^ Moreover, obesity is associated with poor quality of life and disability in people with RA,^44^ which also contributes to the economic burden associated with the disease.

A main limitation of the current study is that obesity is assessed by BMI. As a quantitative measurement, BMI is not a marker of adipose distribution,^45^ and the difference in the ratio between adipose tissue and muscle may account for the adverse effect we see on response to treatment.

In conclusion, we have shown that obesity is associated with a lower likelihood to achieve response to treatment in untreated early RA independently of randomisation arm. Owing to the disease burden associated with these conditions, people with early RA having obesity need a careful follow-up and possibly optimisation of antirheumatic treatments in order to avoid treatment failure.

10.1136/rmdopen-2024-004227.supp2 Supplementary data

## Data Availability

Data are available upon reasonable request. Data are available on reasonable request. Nordic Rheumatic Diseases Strategy Trials and Registries (NORD-STAR) data will not be shared publicly. Access to the NORD-STAR data is organised according to a strict data access procedure. For all types of access, a research proposal must be submitted for evaluation by the NORD-STAR Steering Committee. The evaluation is performed to align the goals of the researchers with the goals of NORD-STAR (which are in turn aligned with the informed consent form signed by NORD-STAR participants). Further information on NORD-STAR data can be obtained by contacting the NORD-STAR Steering Committee (mail to nordstar@ki.se).
