# Supplementary material for: Obesity is a risk factor for poor response to treatment in early rheumatoid arthritis: a NORD-STAR study
Source: RMD Open. 2024 Apr 4;10(2):e004227. doi: 10.1136/rmdopen-2024-004227 (PMC11148705; doi:10.1136/rmdopen-2024-004227)
Supplement: Supplementary data [file rmdopen-2024-004227supp001.pdf]

**Supplementary Table 1.** Baseline characteristics of study participants from the NORD-STAR cohort stratified by treatment arms and BMI.

| Characteristics | Arm 1 | | Arm 2 | | Arm 3 | | Arm 4 | |
|---|---|---|---|---|---|---|---|---|
| | < 30 kg/m$^2$ | ≥ 30 kg/m$^2$ | < 30 kg/m$^2$ | ≥ 30 kg/m$^2$ | < 30 kg/m$^2$ | ≥ 30 kg/m$^2$ | < 30 kg/m$^2$ | ≥ 30 kg/m$^2$ |
| N (%) | 155 (78) | 45 (22) | 165 (81) | 38 (19) | 164 (81) | 39 (19) | 148 (79) | 39 (21) |
| Age, years | 55±15 | 55±12 | 55±16 | 57±12 | 55±15 | 52±12 | 52±15 | 56±11 |
| Women, n (%) | 103 (67) | 36 (80) | 113 (69) | 26 (68) | 112 (68) | 28 (72) | 102 (69) | 27 (69) |
| BMI | 24±3 | 34±4 | 24±3 | 34±4 | 24±3 | 34±3 | 25±3 | 34±4 |
| Current smokers, n (%) | 31 (20) | 4 (9) | 37 (22) | 10 (26) | 41 (25) | 8 (21) | 31 (21) | 11 (28) |
| Ever smokers, n (%) | 92 (59) | 28 (62) | 98 (59) | 28 (74) | 101 (62) | 25 (64) | 78 (53) | 24 (62) |
| RF positive, n (%) | 118 (76) | 33 (73) | 119 (72) | 30 (79) | 129 (79) | 29 (74) | 106 (72) | 28 (72) |
| ACPA positive, n (%) | 127 (82) | 36 (80) | 134 (81) | 32 (84) | 137 (84) | 31 (79) | 118 (80) | 34 (87) |
| Symptom duration, days | 190±148 | 215±219 | 199±159 | 219±197 | 218±173 | 189±142 | 201±150 | 235±173 |
| Time since diagnosis, days | 14±23 | 10±12 | 11±17 | 12±13 | 14±23 | 23.03 | 15±30 | 21±40 |
| ESR, mm/h | 32±25 | 34±26 | 34±25 | 35±20 | 33±26 | 36±31 | 28±20 | 30±15 |
| CRP, mg/L | 23±37 | 24±24 | 22±28 | 15±16 | 20±26 | 18±26 | 19±24 | 17±18 |
| SJC28 | 8±5 | 8±5 | 8±6 | 7±5 | 8±5 | 7±5 | 7±5 | 8±6 |
| SJC66 | 11±8 | 11±6 | 12±8 | 9±7 | 11±7 | 10±8 | 10±6 | 11±8 |
| TJC28 | 10±6 | 11±6 | 9±6 | 9±5 | 9±6 | 10±7 | 8±6 | 11±6 |
| TJC68 | 17±12 | 18±10 | 16±11 | 13±9 | 16±11 | 16±11 | 14±10 | 17±11 |
| Patient Global by VAS (0-100 mm) | 56±24 | 61±21 | 55±24 | 62±24 | 60±24 | 63±24 | 56±23 | 63±20 |
| Patient Pain by VAS (0-100 ml) | 55±24 | 59±24 | 54±24 | 62±25 | 58±25 | 64±21 | 54±22 | 59±25 |
| Physician Global by VAS (0-100 mm) | 49±20 | 50±17 | 49±19 | 52±19 | 52±19 | 52±17 | 49±18 | 54±17 |
| DAS28-ESR | 5.4±1.2 | 5.7±0.9 | 5.4±1.2 | 5.4±1.2 | 5.5±1.1 | 5.4±0.9 | 5.1±1.1 | 5.5±1.1 |
| DAS28-CRP | 5.0±1.1 | 5.3±0.9 | 5.0±1.1 | 4.9±1.1 | 5.0±1.1 | 5.1±0.9 | 4.8±1.0 | 5.2±0.9 |
| SDAI | 30±14 | 32±11 | 30±14 | 29±12 | 30±12 | 31±11 | 28±12 | 32±12 |
| CDAI | 28±13 | 30±10 | 28±13 | 27±11 | 28±11 | 29±11 | 26±11 | 30±12 |

Data are shown as means ± standard deviation for continuous variables and as number (percentage) for categorical variables.

Arm 1 - active conventional treatment (ACT); Arm 2 - methotrexate (MTX) + Certolizumab; Arm 3 - MTX + Abatacept; Arm 4 - MTX + Tocilizumab.

*Abbreviations:* BMI: body mass index, RF: rheumatoid factor, ACPA: anti-cyclic citrullinated peptide, ESR: erythrocyte sedimentation rate, CRP: C-reactive protein, SJC: swollen joint count, TJC: tender joint count, VAS: Visual Analogue Scale, DAS28-ESR: Disease Activity Score with 28 joint using erythrocyte sedimentation rate, DAS28-CRP: Disease Activity Score with 28 joints using C-reactive protein, SDAI: Simplified Disease Activity Index, CDAI: Clinical Disease Activity Index.

**Supplementary Table 2.** Hazard ratios for the achievement of CDAI and SDAI remission and DAS28-CRP<2.6 for BMI>30 mg/kg$^2$.

| | **Unadjusted** | | **Model 1** | | **Model 2** | |
|---|---|---|---|---|---|---|
| | HR (95% CI) | *p*-value | HR (95% CI) | *p*-value | HR (95% CI) | *p*-value |
| CDAI remission | 0.78 (0.62-0.97) | 0.03 | 0.84 (0.67-1.04) | 0.11 | 0.84 (0.67-1.05) | 0.12 |
| SDAI remission | 0.72 (0.58-0.90) | 0.004 | 0.77 (0.61-0.96) | 0.02 | 0.77 (0.62-0.97) | 0.02 |
| DAS28-CRP<2.6 | 0.73 (0.60-0.89) | 0.002 | 0.78 (0.64-0.95) | 0.01 | 0.78 (0.64-0.95) | 0.01 |

Model 1; Adjusted for sex, baseline age, Patient Pain by VAS, and DAS28-CRP.

Model 2; Adjusted for sex, baseline age, current smoking, Patient Pain by VAS, DAS28-CRP, ACPA, and treatment randomization.

*Abbreviations:* HR: hazard ratio, CDAI: Clinical Disease Activity Index, SDAI: Simplified Disease Activity Index, DAS28-CRP: Disease Activity Score with 28 joints using C-reactive protein, VAS: Visual Analogue Scale, ACPA: anti-cyclic citrullinated peptide.

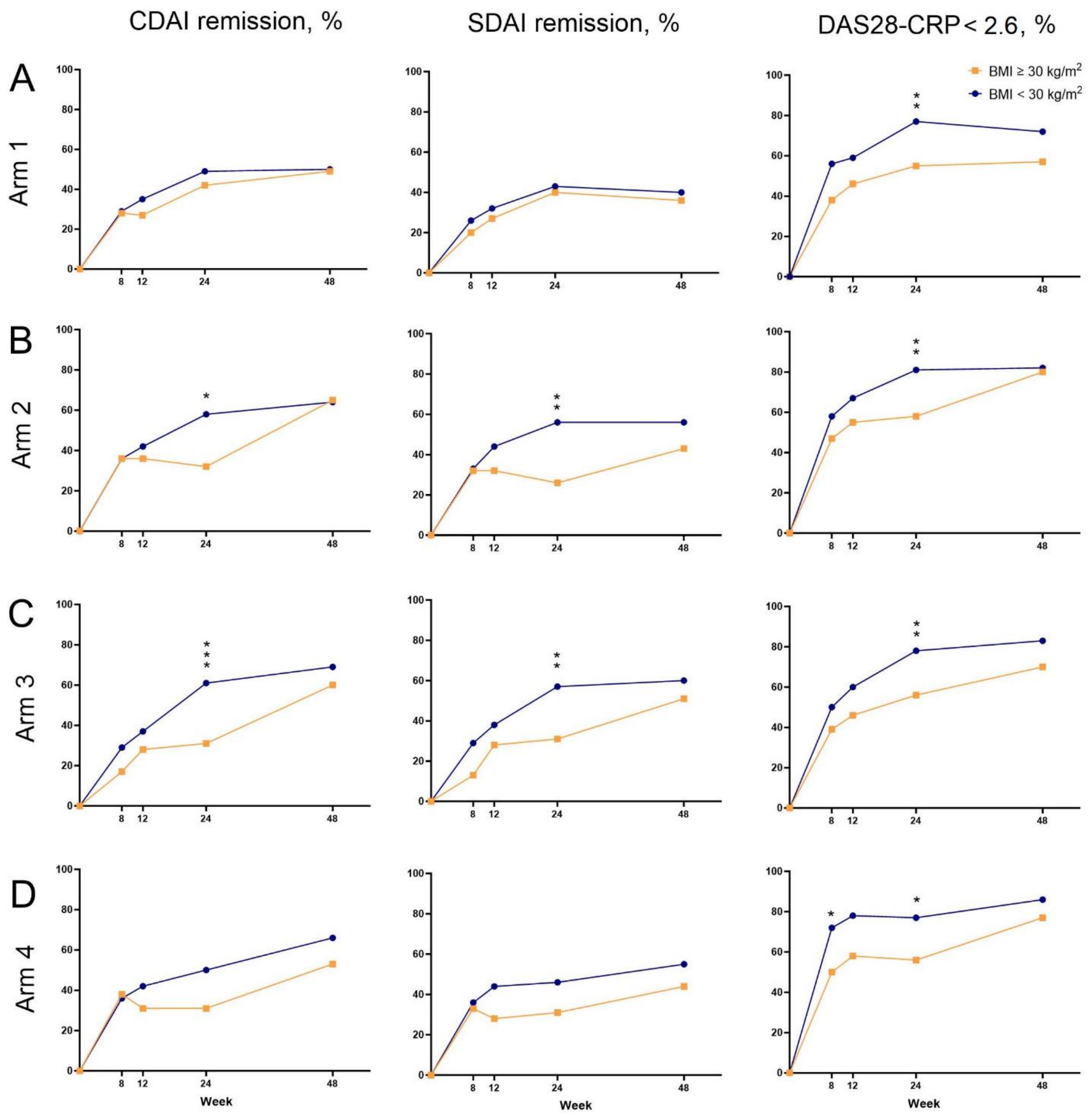


**Supplementary Figure 1.** Response to treatment over time stratified by BMI=30 kg/m$^2$ in the four treatment arms.

Arm 1 - active conventional treatment (ACT; A); Arm 2 - methotrexate (MTX) + Certolizumab (B); Arm 3 - MTX + Abatacept (C); Arm 4 - MTX + Tocilizumab (D).

Response to treatment is shown as % of participants who achieved response to treatment at certain time points during follow-up. *P*-values for remission rates have been calculated by logistic regression analysis adjusted for sex, baseline age, current smoking, Patient Pain by VAS, DAS28-CRP, and ACPA. * $p < 0.05$, ** $p < 0.01$, *** $p < 0.001$.

*Abbreviations:* RA: rheumatoid arthritis, BMI: body mass index, CDAI: Clinical Disease Activity Index, SDAI: Simplified Disease Activity Index, DAS28-CRP: Disease Activity Score with 28 joints using C-reactive protein.

**Supplementary Table 3.** Hazard ratios for response to treatment (CDAI remission, SDAI remission, and DAS28-CRP<2.6) for BMI>30 mg/kg$^2$ stratified by treatment arm.

| | | **Unadjusted** | | **Model 1** | | **Model 2** | |
|---|---|---|---|---|---|---|---|
| | | HR (95% CI) | *p*-value | HR (95% CI) | *p*-value | HR (95% CI) | *p*-value |
| **Arm 1** | CDAI | 0.83 (0.53-1.30) | 0.42 | 0.96 (0.60-1.52) | 0.85 | 0.96 (0.60-1.52) | 0.85 |
| | SDAI | 0.74 (0.46-1.18) | 0.20 | 0.84 (0.52-1.34) | 0.46 | 0.82 (0.51-1.32) | 0.41 |
| | DAS28-CRP | 0.69 (0.46-1.02) | 0.07 | 0.76 (0.51-1.14) | 0.18 | 0.79 (0.53-1.19) | 0.26 |
| **Arm 2** | CDAI | 0.92 (0.60-1.42) | 0.71 | 0.93 (0.60-1.44) | 0.74 | 0.95 (0.61-1.47) | 0.81 |
| | SDAI | 0.80 (0.52-1.24) | 0.32 | 0.80 (0.52-1.25) | 0.34 | 0.82 (0.52-1.28) | 0.38 |
| | DAS28-CRP | 0.73 (0.49-1.10) | 0.13 | 0.76 (0.50-1.14) | 0.19 | 0.75 (0.50-1.14) | 0.18 |
| **Arm 3** | CDAI | 0.68 (0.44-1.05) | 0.08 | 0.71 (0.46-1.10) | 0.12 | 0.71 (0.46-1.11) | 0.13 |
| | SDAI | 0.67 (0.45-1.05) | 0.08 | 0.71 (0.46-1.09) | 0.11 | 0.71 (0.46-1.09) | 0.12 |
| | DAS28-CRP | 0.79 (0.53-1.17) | 0.24 | 0.78 (0.52-1.16) | 0.22 | 0.77 (0.52-1.15) | 0.20 |
| **Arm 4** | CDAI | 0.71 (0.45-1.12) | 0.14 | 0.82 (0.51-1.32) | 0.41 | 0.84 (0.52-1.36) | 0.47 |
| | SDAI | 0.68 (0.43-1.07) | 0.10 | 0.78 (0.48-1.25) | 0.30 | 0.78 (0.48-1.27) | 0.32 |
| | DAS28-CRP | 0.75 (0.50-1.12) | 0.16 | 0.83 (0.55-1.25) | 0.37 | 0.82 (0.54-1.25) | 0.35 |

Model 1; Adjusted for sex, baseline age, Patient Pain by VAS, and DAS28-CRP.

Model 2; Adjusted for sex, baseline age, current smoking, Patient Pain by VAS, DAS28-CRP, ACPA.

Arm 1 - active conventional treatment (ACT); Arm 2 - methotrexate (MTX) + Certolizumab; Arm 3 - MTX + Abatacept; Arm 4 - MTX + Tocilizumab.

*Abbreviations:* HR: hazard ratio, CDAI, Clinical Disease Activity Index, DAS28-CRP: Disease Activity Score with 28 joints using C-reactive protein, SDAI: Simplified Disease Activity Index, ACPA: anti-cyclic citrullinated peptide.

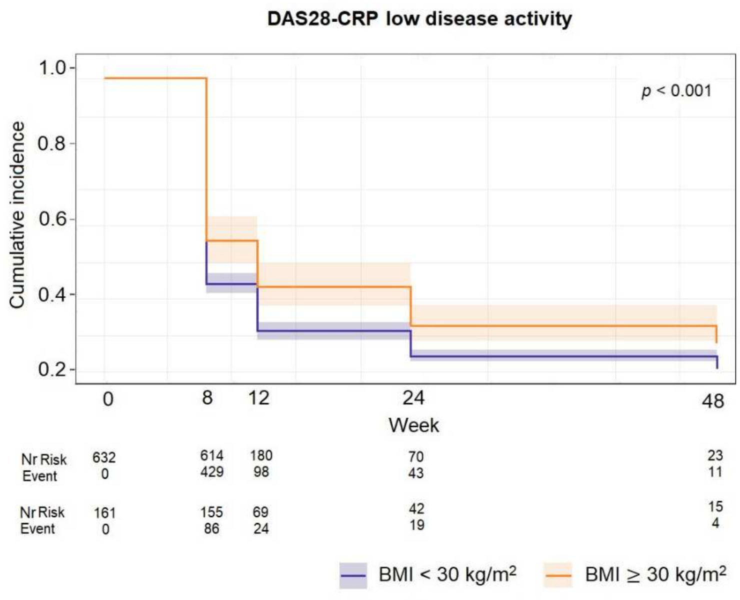


**Supplementary Figure 2.** Kaplan-Meier curves for DAS28-CRP low disease activity.

*Abbreviations:* BMI: body mass index, DAS28-CRP: Disease Activity Score with 28 joints using C-reactive protein.

**Supplementary Table 4.** Hazard ratios for DAS28-CRP low disease activity for BMI >30 mg/kg$^2$.

| | Unadjusted | | Model 1 | | Model 2 | |
|---|---|---|---|---|---|---|
| | HR (95% CI) | *p*-value | HR (95% CI) | *p*-value | HR (95% CI) | *p*-value |
| DAS28-CRP low disease activity | 0.75 (0.62-0.90) | 0.003 | 0.78 (0.64-0.94) | 0.01 | 0.78 (0.64-0.95) | 0.01 |

Model 1; Adjusted for sex, baseline age, Patient Pain by VAS, and DAS28-CRP.

Model 2; Adjusted for sex, baseline age, current smoking, Patient Pain by VAS, DAS28-CRP, ACPA, and treatment randomization.

*Abbreviations:* DAS28-CRP: Disease Activity Score with 28 joints using C-reactive protein, HR: hazard ratio, VAS: Visual Analogue Scale, ACPA: anti-cyclic citrullinated peptide.

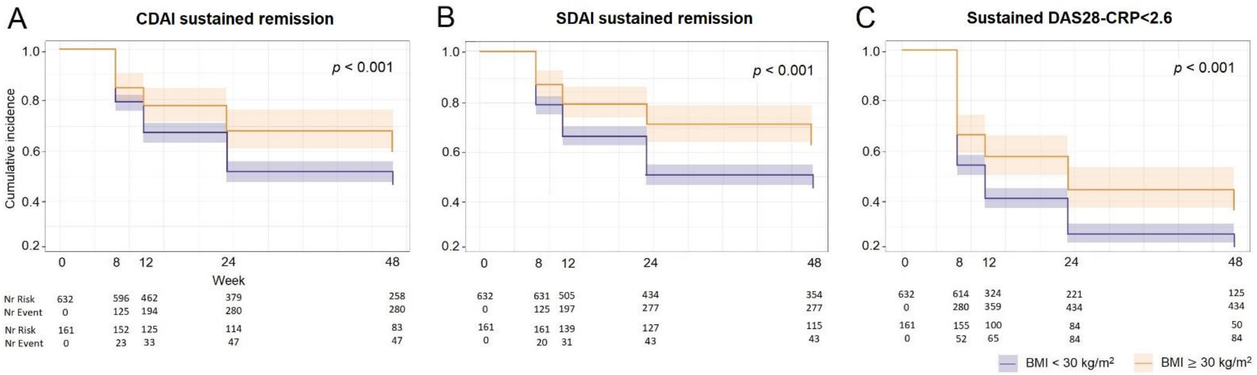


**Supplementary Figure 3.** Kaplan-Meier curves for sustained response to treatment stratified by BMI=30 kg/m$^2$.

CDAI sustained remission (A); SDAI sustained remission (B); sustained DAS28-CRP<2.6 (C).

*Abbreviations:* BMI: body mass index, CDAI: Clinical Disease Activity Index, SDAI: Simplified Disease Activity Index, DAS28-CRP: Disease Activity Score with 28 joints using C-reactive protein.

**Supplementary Table 5.** Hazard ratios for sustained response to treatment for BMI >30 mg/kg$^2$.

| | Unadjusted | | Model 1 | | Model 2 | |
|---|---|---|---|---|---|---|
| | HR (95% CI) | *p*-value | HR (95% CI) | *p*-value | HR (95% CI) | *p*-value |
| CDAI sustained remission | 0.62 (0.45-0.84) | 0.002 | 0.68 (0.50-0.93) | 0.02 | 0.68 (0.50-0.93) | 0.02 |
| SDAI sustained remission | 0.57 (0.42-0.79) | <0.001 | 0.63 (0.45-0.87) | 0.01 | 0.63 (0.46-0.87) | 0.01 |
| DAS28-CRP<2.6, sustained | 0.68 (0.54-0.86) | 0.001 | 0.73 (0.58-0.92) | 0.01 | 0.73 (0.58-0.92) | 0.01 |

Model 1; Adjusted for sex, baseline age, Patient Pain by VAS, and DAS28-CRP.

Model 2; Adjusted for sex, baseline age, current smoking, Patient Pain by VAS, DAS28-CRP, ACPA, and treatment randomization.

Abbreviations: HR: hazard ratio, CDAI: Clinical Disease Activity Index, SDAI: Simplified Disease Activity Index, DAS28-CRP: Disease Activity Score with 28 joints using C-reactive protein, VAS: Visual Analogue Scale, ACPA: anti-cyclic citrullinated peptide.

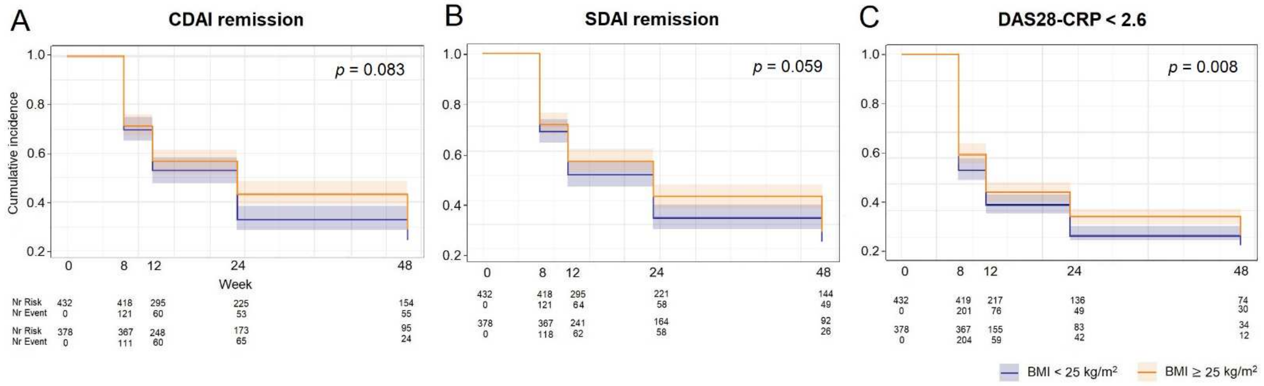


**Supplementary Figure 4.** Kaplan-Meier curves for CDAI and SDAI remission and DAS28-CRP<2.6 stratified by BMI=25 kg/m$^2$.

CDAI remission (A); SDAI remission (B); DAS28-CRP < 2.6 (C).

*Abbreviations:* BMI: body mass index, CDAI: Clinical Disease Activity Index, DAS28-CRP: Disease Activity Score with 28 joints using C-reactive protein, SDAI: Simplified Disease Activity Index.

**Supplementary Table 6.** Hazard ratios for remission stratified by BMI >25 kg/m$^2$.

| | **Unadjusted** | | **Model 1** | | **Model 2** | |
|---|---|---|---|---|---|---|
| | HR (95% CI) | *p*-value | HR (95% CI) | *p*-value | HR (95% CI) | *p*-value |
| CDAI remission | 0.88 (0.74-1.04) | 0.14 | 0.92 (0.77-1.09) | 0.34 | 0.91 (0.76-1.08) | 0.26 |
| SDAI remission | 0.87 (0.74-1.03) | 0.11 | 0.91 (0.77-1.08) | 0.29 | 0.89 (0.75-1.06) | 0.20 |
| DAS28-CRP<2.6 | 0.86 (0.74-1.00) | 0.05 | 0.89 (0.76-1.04) | 0.14 | 0.88 (0.75-1.04) | 0.12 |

Model 1; Adjusted for sex, baseline age, Patient Pain by VAS, and DAS28-CRP.

Model 2; Adjusted for sex, baseline age, current smoking, Patient Pain by VAS, DAS28-CRP, ACPA, and treatment randomization.

*Abbreviations:* BMI: body mass index, HR: hazard ratio, CDAI: Clinical Disease Activity Index, SDAI: Simplified Disease Activity Index, DAS28-CRP: Disease Activity Score with 28 joints using C-reactive protein, VAS: Visual Analogue Scale, ACPA: anti-cyclic citrullinated peptide.

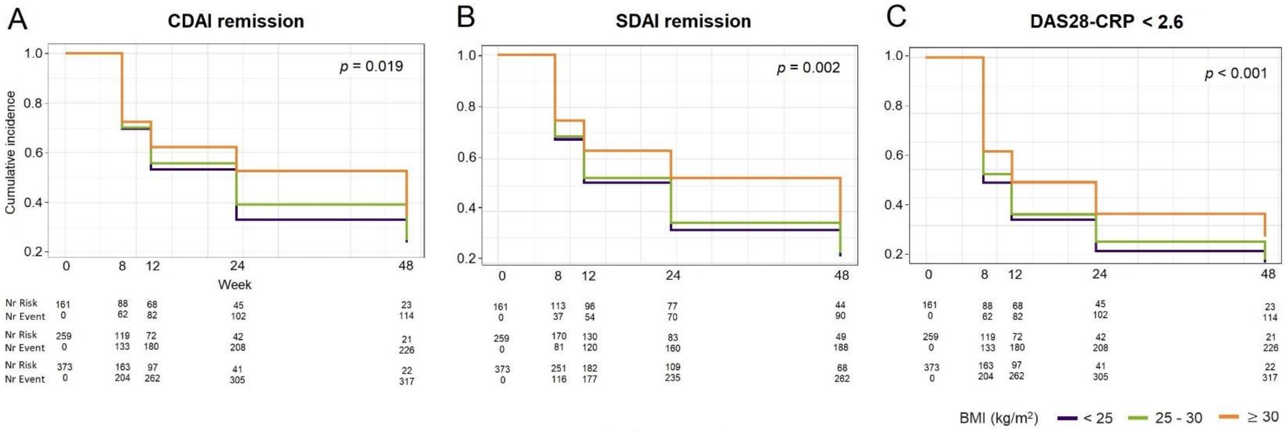


**Supplementary Figure 5.** Kaplan-Meier curves for CDAI and SDAI remission and DAS28-CRP<2.6 for three BMI groups: normal weight (≤25 kg/m$^2$), overweight (25-30 kg/m$^2$), and obesity (≥30 kg/m$^2$).

CDAI remission (A); SDAI remission (B); DAS28-CRP <2.6 (C).

*Abbreviations:* BMI: body mass index, CDAI: Clinical Disease Activity Index, SDAI: Simplified Disease Activity Index; DAS28-CRP: Disease Activity Score with 28 joints using C-reactive protein.

**Supplementary Table 7.** Hazard ratios for remission scores for three BMI classes (BMI<25 kg/m$^2$ as reference).

| | BMI (kg/m$^2$) | Unadjusted | | Model 1 | | Model 2 | |
|---|---|---|---|---|---|---|---|
| | | HR (95% CI) | *p*-value | HR (95% CI) | *p*-value | HR (95% CI) | *p*-value |
| CDAI remission | <25 | Ref. | ·· | Ref. | ·· | Ref. | ·· |
| | 25-30 | 0.96 (0.79-1.16) | 0.66 | 0.98 (0.80-1.19) | 0.82 | 0.96 (0.79-1.17) | 0.68 |
| | ≥30 | 0.76 (0.60-0.97) | 0.02 | 0.83 (0.65-1.05) | 0.12 | 0.82 (0.65-1.04) | 0.11 |
| SDAI remission | <25 | Ref. | ·· | Ref. | ·· | Ref. | ·· |
| | 25-30 | 0.98 (0.81-1.18) | 0.83 | 1.01 (0.83-1.22) | 0.95 | 0.98 (0.81-1.19) | 0.84 |
| | ≥30 | 0.72 (0.56-0.91) | 0.005 | 0.77 (0.61-0.98) | 0.03 | 0.77 (0.60-0.97) | 0.03 |
| DAS28-CRP<2.6 | <25 | Ref. | ·· | Ref. | ·· | Ref. | ·· |
| | 25-30 | 0.96 (0.81-1.14) | 0.61 | 0.97 (0.82-1.16) | 0.76 | 0.97 (0.81-1.15) | 0.69 |
| | ≥30 | 0.72 (0.58-0.89) | 0.002 | 0.77 (0.62-0.95) | 0.02 | 0.77 (0.62-0.95) | 0.02 |

Model 1; Adjusted for sex, baseline age, Patient Pain by VAS, and DAS28-CRP.

Model 2; Adjusted for sex, baseline age, current smoking, Patient Pain by VAS, DAS28-CRP, ACPA, and treatment randomization.

*Abbreviations:* HR: hazard ratio, CDAI: Clinical Disease Activity Index, SDAI: Simplified Disease Activity Index, DAS28-CRP: Disease Activity Score with 28 joints using C-reactive protein, VAS: Visual Analogue Scale, ACPA: anti-cyclic citrullinated peptide.

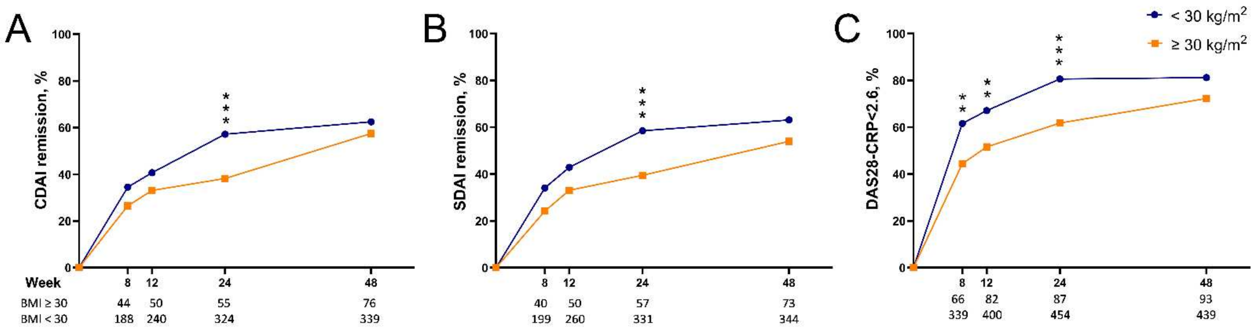


**Supplementary Figure 6.** Remission over time stratified by BMI=30 kg/m$^2$ in the per-protocol population.

CDAI remission (A); SDAI remission (B); DAS28-CRP <2.6 (C).

Remission rates are shown as % of participants who achieved remission at any time point during follow-up. *P*-values for remission rates have been calculated by logistic regression analysis adjusted for sex, baseline age, current smoking, Patient Pain by VAS, DAS28-CRP, ACPA, and treatment randomization. * $p < 0.05$, ** $p < 0.01$, *** $p < 0.001$.

*Abbreviations:* BMI: body mass index, CDAI: Clinical Disease Activity Index, SDAI: Simplified Disease Activity Index DAS28-CRP: Disease Activity Score with 28 joints using C-reactive protein.

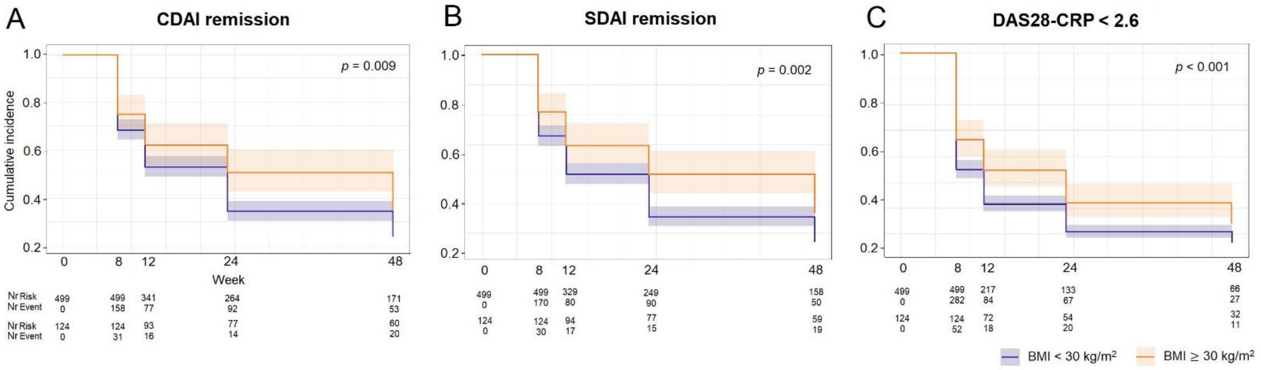


**Supplementary Figure 7.** Kaplan-Meier curves for CDAI and SDAI remission and DAS28-CRP<2.6 in the per-protocol population stratified for BMI=30 kg/m$^2$.

CDAI remission (A); SDAI remission (B); DAS28-CRP <2.6 (C).

*Abbreviations:* BMI: body mass index, CDAI: Clinical Disease Activity Index, SDAI: Simplified Disease Activity Index, DAS28-CRP: Disease Activity Score with 28 joints using C-reactive protein.

**Supplementary Table 8.** Hazard ratios for remission in the per-protocol population stratified for BMI=30 kg/m$^2$.

| | **Unadjusted** | | **Model 1** | | **Model 2** | |
|---|---|---|---|---|---|---|
| | HR (95% CI) | *p*-value | HR (95% CI) | *p*-value | HR (95% CI) | *p*-value |
| CDAI remission | 0.77 (0.60-0.97) | 0.03 | 0.83 (0.65-1.05) | 0.12 | 0.82 (0.64-1.05) | 0.11 |
| SDAI remission | 0.72 (0.57-0.92) | 0.01 | 0.78 (0.61-1.00) | 0.04 | 0.77 (0.60-0.98) | 0.04 |
| DAS28-CRP <2.6 | 0.73 (0.59-0.91) | 0.004 | 0.77 (0.62-0.96) | 0.02 | 0.77 (0.62-0.95) | 0.02 |

Model 1; Adjusted for sex, baseline age, Patient Pain by VAS, and DAS28-CRP.

Model 2; Adjusted for sex, baseline age, current smoking, Patient Pain by VAS, DAS28-CRP, ACPA, and treatment randomization.

*Abbreviations:* HR: hazard ratio, CDAI: Clinical Disease Activity Index, SDAI: Simplified Disease Activity Index, DAS28-CRP: Disease Activity Score with 28 joints using C-reactive protein, VAS: Visual Analogue Scale, ACPA: anti-cyclic citrullinated peptide.
